# Accuracy of cognitive vs software‐guided MRI‐targeted biopsy in predicting final grading at prostatectomy

**DOI:** 10.1111/bju.16903

**Published:** 2025-08-22

**Authors:** Marco Finati, Anna Ricapito, Gennaro Musi, Bernardo Rocco, Francesco Porpiglia, Alessandro Antonelli, Paolo Gontero, Peter Bostrom, Luigi Schips, Giuseppe Simone, Vincenzo Mirone, Luca Carmignani, Giuseppe Mario Lodovico, Emanuele Montanari, Alessandro Sciarra, Pierluigi Bove, Ivan Jambor, Carlo Trombetta, Vincenzo Ficarra, Giuseppe Carrieri, Ugo Giovanni Falagario, PROMOD Working Group

**Affiliations:** ^1^ Department of Urology and Renal Transplantation University of Foggia Foggia Italy; ^2^ Department of Urology IEO European Institute of Oncology, IRCCS Milan Italy; ^3^ Department of Urology, ‘A. Gemelli’ Hospital Foundation IRCCS, Università Cattolica del Sacro Cuore Rome Italy; ^4^ Department of Urology, Azienda Ospedaliera Universitaria ‘San Luigi Gonzaga’ University of Turin Turin Italy; ^5^ Department of Urology Azienda Ospedaliera Universitaria Integrata di Verona, University of Verona Verona Italy; ^6^ Department of Surgical Sciences Città della Salute e della Scienza di Torino, Molinette Hospital Turin Italy; ^7^ Department of Urology University of Turku and Turku University Hospital Turku Finland; ^8^ Department of Urology University ‘G.d'Annunzio’ Chieti‐Pescara Italy; ^9^ Department of Oncologic Urology IRCCS ‘Regina Elena’ National Cancer Institute of Rome Rome Italy; ^10^ Department of Neurosciences, Reproductive Sciences and Odontostomatology University of Naples ‘Federico II’ Naples Italy; ^11^ Department of Urology IRCCS Policlinico San Donato Milan Italy; ^12^ Department of Urology Ente Ecclesiastico Miulli Acquaviva delle Fonti Italy; ^13^ Department of Urology IRCCS Foundation Ca' Granda ‐ Maggiore Policlinico Hospital Milan Italy; ^14^ Department of Maternal Infant and Urologic Sciences ‘Sapienza’ University of Rome Rome Italy; ^15^ Department of Urology San Carlo di Nancy Hospital Rome Italy; ^16^ Department of Radiology University of Turku and Medical Imaging Centre of Southwest Finland, Turku University Hospital Turku Finland; ^17^ Department of Medicine, Surgery and Health Sciences University of Trieste Trieste Italy; ^18^ Department of Urology University of Messina Messina Italy; ^19^ Urology Unit, department of Molecular Medicine and Surgery, (Solna) Karolinska Institutet Stockholm Sweden

**Keywords:** prostatic neoplasms, diagnosis, magnetic resonance imaging, target biopsy, Detection rate

**Abbreviations**
GG: Grade Group
EAU: European Association of Urology
ISUP: International Society of Urological Pathology
PCa: prostate cancer
PI‐RADS: Prostate Imaging–Reporting and Data System
RP: radical prostatectomy
SBx: systematic biopsy
TBx: targeted biopsy

The FUTURE randomised trial, which included patients with prior negative prostate biopsy undergoing MRI targeted biopsy (TBx), found no superiority among cognitive, in‐bore and software‐guided techniques in detecting prostate cancer (PCa) [1]. These findings were confirmed by recent meta‐analyses [2, 3], leading the European Association of Urology (EAU) to refrain from recommending any one MRI‐TBx technique over another in their PCa guidelines [4]. However, no study has compared these techniques against final histopathological examination after radical prostatectomy (RP) to determine if any one of them better predicts PCa risk assessment before radical surgery. In this study, we aimed to evaluate the cognitive approach to MRI‐TBx vs software‐guided MRI‐TBx to determine which technique better predicts final pathology at RP.

A total of 9966 patients were identified from the PROMOD study dataset (NCT05078359 – IRB University of Foggia: 143/CE/2020, DDG n. 696), a retrospective international observational study enrolling institutions performing TBx, with the aim of exploring inter‐centre differences in the accuracy of MRI and defining optimal strategies for TBx [5]. We included only patients with positive MRI (Prostate Imaging–Reporting and Data System [PI‐RADS] score >2; *n* = 1557 excluded), diagnosed with PCa on combined systematic biopsy (SBx) + TBx (*n* = 2910), who subsequently underwent RP (*n* = 1991) between 2015 and 2022. All MRI scans were acquired and interpreted at the centre where the TBx was performed. PI‐RADS v2.0 was used for interpretation, with v2.1 adopted from 2019 onwards. Patients with a PSA level ≥ 50 ng/mL (*n* = 30), those who had previously undergone prostate surgery (*n* = 28), and those who underwent TBx with combined cognitive/software‐guided technique or in‐bore TBx (*n* = 2405) were also excluded. The final cohort included 1045 patients, of whom 327 (31%) underwent TBx using the cognitive approach and 718 (69%) underwent software‐guided TBx (Fig. S1). The endpoints were histopathological concordance/discordance between TBx and RP, defined as any difference in International Society of Urological Oncology (ISUP) Grade Group (GG) between targeted cores and RP. Concordance/Discordance between the two approaches were also evaluated using  overall ISUP GG from TBx + SBx as reference. Distributions of ISUP GG concordance, upgrading or downgrading (including missed cancer at TBx) were reported as percentage and proportions and tested with chi‐squared or McNemar tests, as appropriate. Multivariate logistic regression analysis was performed to evaluate the impact of the cognitive vs the software‐guided TBx approach on histopathological concordance between TBx and RP, after adjusting for all available covariates. We then performed 1:1 propensity‐score matching to create a matched cohort equally distributed according to TBx technique (software‐guided vs cognitive). The greedy nearest‐neighbour method, with a calliper of 0.1, was used to create the matched cohort, while standardised mean differences were calculated to assess the quality of matching distribution among all clinicopathological variables (Table S1) [6]. The same logistic regression was then repeated in the matched cohort. All statistical analyses were performed using RStudio© (Rstudio Team, 2023.6.1.524).

The median (interquartile range) age of our cohort was 66 (61–71) years. The clinicopathological characteristics of our cohort are described in Table S1. For combined SBx + TBx, ISUP GG concordance, upgrading and downgrading at RP were 60%, 25% and 15%, respectively, for the software‐guided TBx group, vs 52%, 31% and 17%, respectively, for the cognitive TBx group. When comparing ISUP GG at RP with TBx only, software‐guided TBx cores showed concordance, upgrading and downgrading rates of 52%, 39% and 9%, respectively, vs 48%, 40% and 12%, respectively, in the cognitive TBx group (Fig. S2). Cross‐tabulation of the highest GG detected at TBx and final GG at RP is reported in Table S2, stratified by TBx fusion method. The propensity‐score matching yielded a cohort of 540 patients equally distributed between the cognitive and software‐guided techniques. Standardised mean difference did not differ by more than 0.1 for all the covariates included in the matching, indicating a well‐balanced match [7] (Table S1). In the matched cohort, ISUP GG concordance, upgrading and downgrading rates at TBx were 55%, 37% and 8%, respectively, for the software‐guided approach, vs 48%, 39% and 13%, respectively, for the cognitive approach (Fig. 1). Adding SBx to TBx significantly improved concordance (56% vs 52%; *P* = 0.001) and reduced upgrading (31% vs 38%), regardless of TBx fusion technique used (Fig. 1). Multivariate logistic regression analysis (Table S3) showed no significant association between TBx fusion method and histopathological concordance, whether comparing TBx alone or SBx + TBx with final RP pathology. Conversely, the transrectal approach was associated with a lower concordance ratio, but only when considering ISUP GG from SBx + TBx (hazard ratio 0.69, 95% CI 0.48–0.98; *P* = 0.04 [Table S3]). Results from the multivariable analysis of ISUP upgrading at RP (Table S4) were consistent with these concordance‐based findings.

**Fig. 1:**
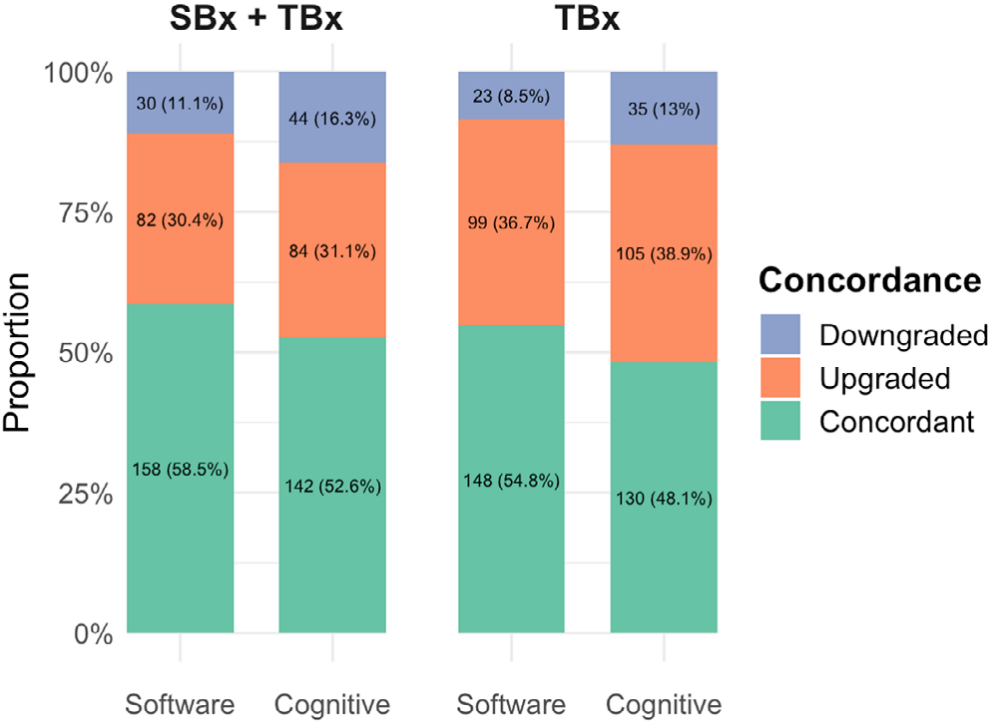
Bar chart showing pathological concordance results according to prostate biopsy and radical prostatectomy results in the matched cohort. SBx, systematic biopsy; TBx, targeted biopsy.

Emerging evidence is shifting the diagnostic strategy for prostate biopsy toward combining TBx with perilesional sampling [8], while reducing reliance on SBx cores [4]. In this context, optimising TBx sampling is crucial to accurately assess PCa risk category before radical surgery.

To our knowledge, this study is the first to demonstrate that neither the cognitive nor the software‐guided TBx technique is superior in predicting final pathology at RP. While EAU PCa guidelines currently do not favour one TBx technique over another based on no difference in PCa detection rates, our findings reinforce this recommendation using definitive ISUP GG as a reference. However, the overall pathological concordance observed remained low, warranting cautious interpretation. Despite no observed differences in histopathological concordance, significant differences in ISUP GG and risk group migration emerged between the techniques. Specifically, cognitive TBx was associated with a higher risk of ISUP GG upstaging from GG 1 to GG 2 (82% vs 63%) and missed 8.3% of all PCa and 7.0% of clinically significant PCa (ISUP GG ≥2), compared to 10.3% and 4.6%, respectively, for software‐guided TBx. Similarly, 79% of EAU low‐risk cases in the cognitive TBx group progressed to intermediate risk at final pathology, vs 66% for software‐guided TBx, a disparity even amplified after propensity‐score adjustment (Table S5). These findings indicate that patients undergoing cognitive TBx faced a higher risk of upstaging at RP, despite similar overall histopathological concordance between the two fusion techniques.

This study has some limitations. While our propensity‐score model accounted for all clinical, pathological and radiological variables and achieved excellent matching distribution, the retrospective nature of the cohort and lack of centralised MRI review introduce potential biases. Data on operator experience, the specific fusion software used, and whether lesion contouring was performed by radiologists or urologists were unavailable. These factors may have influenced both MRI interpretation and prostate biopsy accuracy, as previously highlighted in the literature [9].

In conclusion, our findings confirm that, even when compared with final pathology at RP, software‐guided and cognitive TBx approaches yield no significant differences in predicting final ISUP GGs. However, differences in ISUP GG and risk group migration suggest that software‐guided TBx might improve pre‐surgical risk assessment, while adding SBx is still preferred to better predict the final pathological grading at RP.

## Funding

None.

## Disclosure of Interests

The authors have no relevant declarations of interest.

## Supporting information


**Fig. S1.** Consolidated Standards of Reporting Trials diagram with selection criteria.
**Fig. S2.** Bar chart showing pathological concordance results according to prostate biopsy and radical prostatectomy results in the overall unmatched cohort. SBx, systematic biopsy; TBx, targeted biopsy.
**Table S1.** Descriptive Statistics of the overall cohort and a Propensity Score Matched group of 540 Patients equally distributed for Targeted Biopsy Technique (Software‐guided vs Cognitive).
**Table S2.** Cross‐tabulation of highest International Society of Urological Pathology (ISUP) grade group found at Targeted Biopsy (TBx) and final grade at prostatectomy, stratified for TBx technique.
**Table S3.** Multivariable logistic regression on International society of Urological Pathology concordance at radical prostatectomy in the matched cohort, compared to ISUP grade at targeted biopsy (TBx) and Systematic + Targeted Biopsy (SBx + TBx).
**Table S4.** Multivariable logistic regression on International society of Urological Pathology upgrade at radical prostatectomy in the matched cohort, compared to ISUP grade at targeted biopsy (TBx) and Systematic + Targeted Biopsy (SBx + TBx).
**Table S5.** Cross‐tabulation of European Association of Urology (EAU) prostate cancer risk group at Targeted Biopsy (TBx) and final risk group at prostatectomy, stratified for TBx technique.
