# Supplementary material for: Accuracy of cognitive vs software‐guided MRI‐targeted biopsy in predicting final grading at prostatectomy
Source: BJU Int. 2025 Aug 22;136(6):1017–9. doi: 10.1111/bju.16903 (PMC12606516; doi:10.1111/bju.16903)
Supplement: Supplementary file 1 — Fig. S1. Consolidated Standards of Reporting Trials diagram with selection criteria. Fig. S2. Bar chart showing pathological concordance results according to prostate biopsy and radical prostatectomy results in the overall unmatched cohort. SBx, systematic biopsy; TBx, targeted biopsy. Table S1. Descriptive Statistics of the overall cohort and a Propensity Score Matched group of 540 Patients equally distributed for Targeted Biopsy Technique (Software‐guided vs Cognitive). Table S2. Cross‐tabulation of highest International Society of Urological Pathology (ISUP) grade group found at Targeted Biopsy (TBx) and final grade at prostatectomy, stratified for TBx technique. Table S3. Multivariable logistic regression on International society of Urological Pathology concordance at radical prostatectomy in the matched cohort, compared to ISUP grade at targeted biopsy (TBx) and Systematic + Targeted Biopsy (SBx + TBx). Table S4. Multivariable logistic regression on International society of Urological Pathology upgrade at radical prostatectomy in the matched cohort, compared to ISUP grade at targeted biopsy (TBx) and Systematic + Targeted Biopsy (SBx + TBx). Table S5. Cross‐tabulation of European Association of Urology (EAU) prostate cancer risk group at Targeted Biopsy (TBx) and final risk group at prostatectomy, stratified for TBx technique. [file BJU-136-1017-s001.docx]

**Supplementary Figure 1.** Consolidated Standards of Reporting Trials diagram with selection criteria.


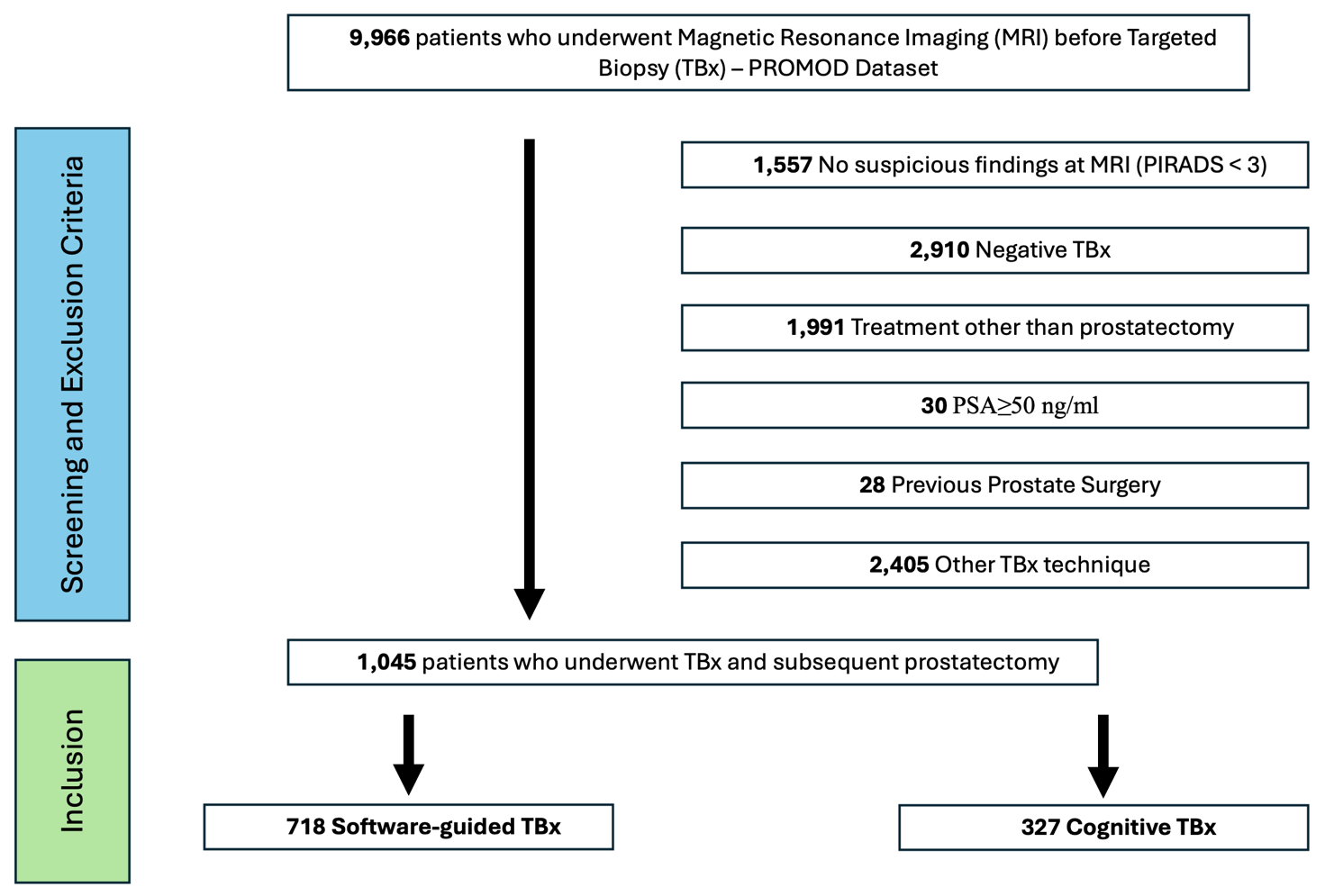


**Supplementary Figure 2.** Bar chart showing pathological concordance results according to prostate biopsy and radical prostatectomy results in the overall unmatched cohort. SBx = systematic biopsy; TBx: targeted biopsy.


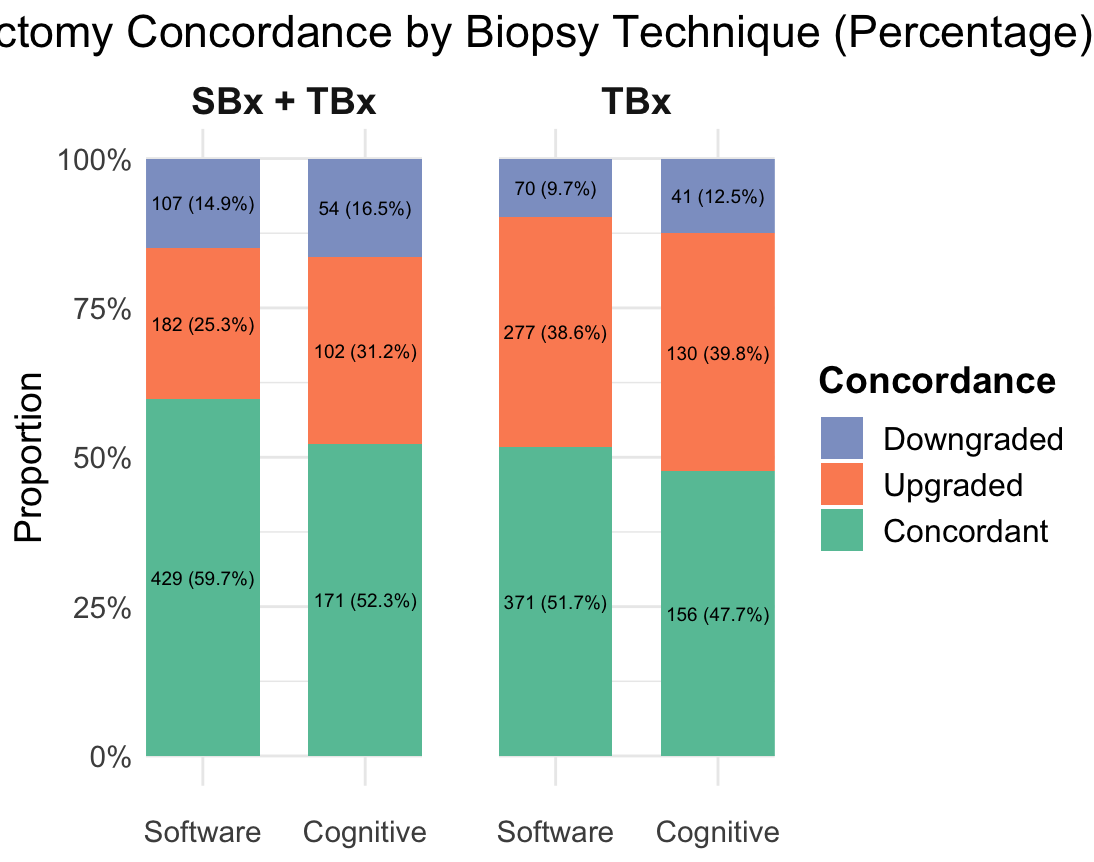


**Supplementary Table 1.** Descriptive Statistics of the overall cohort and a Propensity Score Matched group of 540 Patients equally distributed for Targeted Biopsy Technique (Software-guided versus Cognitive).

|  | Unmatched Cohort | | Matched Cohort | | |
| --- | --- | --- | --- | --- | --- |
| TBx Technique | Software-guided  (N=718) | Cognitive  (N=327) | Software-guided  (N=270) | Cognitive  (N=270) | Standardized Mean Difference (SMD)* |
| Age, years |  |  |  |  | 0.032 |
| Median (IQR) | 67 (61,71) | 66 (60,69) | 66 (60,69) | 66 (61,70) |  |
| PSA, ng/ml |  |  |  |  | 0.036 |
| Median (IQR) | 6.50 (5.00, 9.22) | 7.15 (5.22, 9.35) | 6.64 (5.21, 9.29) | 7.27 (5.21, 9.40) |  |
| Prostate Volume, cc |  |  |  |  | 0.068 |
| Median (IQR) | 45 (34,55) | 40 (30,52) | 43 (33,52) | 41 (31,53) |  |
| Maximum MRI lesion diameter, mm |  |  |  |  | 0.097 |
| Median (IQR) | 5 (2-14) | 9 (4, 20) | 8 (3, 18) | 9 (3, 18) |  |
| MRI Scanner, Tesla |  |  |  |  | 0.004 |
| 1.5 | 526 (73%) | 128 (39%) | 125 (46.%) | 127 (47%) |  |
| 3 | 192 (27%) | 199 (61%) | 145 (54%) | 143 (53%) |  |
| Prostate biopsy history: |  |  |  |  | 0.007 |
| Previous negative | 118 (16%) | 22 (6.7%) | 23 (8.5%) | 21 (7.8%) |  |
| Biopsy Naive | 600 (84%) | 305 (93%) | 247 (91.5%) | 249 (92.2%) |  |
|  |  |  |  |  |  |
| DRE findings, n (%): |  |  |  |  | 0.004 |
| Negative | 464 (65%) | 185 (57%) | 156 (58%) | 158 (59%) |  |
| Positive/Suspicious | 254 (35%) | 142 (43%) | 114 (42%) | 112 (41%) |  |
| Highest PIRADS Score, n (%) |  |  |  |  | 0.015 |
| 3 | 117 (16%) | 29 (8.9%) | 31 (12%) | 29 (11%) |  |
| 4 | 423 (59%) | 118 (36%) | 117 (43%) | 116 (43%) |  |
| 5 | 178 (25%) | 180 (55%) | 122 (45%) | 125 (46%) |  |
| Biopsy Approach: |  |  |  |  | 0.026 |
| Transrectal | 402 (56%) | 221 (67%) | 157 (58%) | 165 (61%) |  |
| Transperineal | 316 (44%) | 106 (32%) | 113 (42%) | 105 (39%) |  |
| ISUP grade at SBx+TBx, n (%): |  |  |  |  | 0.099 |
| 1 | 178 (25%) | 40 (12%) | 42 (16%) | 36 (13%) |  |
| 2 | 307 (43%) | 132 (40%) | 123 (46%) | 112 (42%) |  |
| 3 | 116 (16%) | 82 (25%) | 45 (17%) | 66 (24%) |  |
| 4 | 83 (12%) | 46 (14%) | 41 (15%) | 36 (13%) |  |
| 5 | 34 (4.7%) | 27 (8.3%) | 19 (7.0%) | 20 (7.4%) |  |
| ISUP grade at TBx, n (%) |  |  |  |  |  |
| No Cancer | 74 (10%) | 27 (8%) | 15 (5.6%) | 22 (8.1%) |  |
| 1 | 167 (23%) | 39 (12%) | 42 (16%) | 34 (13%) |  |
| 2 | 285 (40%) | 124 (38%) | 122 (45%) | 103 (38%) |  |
| 3 | 105 (15%) | 77 (24%) | 40 (15%) | 64 (24%) |  |
| 4 | 59 (8.2%) | 36 (11%) | 33 (12%) | 30 (11%) |  |
| 5 | 28 (3.9%) | 24 (7.3%) | 18 (6.7%) | 17 (6.3%) |  |
| Clinical T Stage, n (%): |  |  |  |  |  |
| 1 | 312 (44%) | 120 (37%) | 95 (35%) | 110 (41%) |  |
| 2 | 373 (52%) | 180 (55%) | 156 (58%) | 142 (53%) |  |
| 3 | 33 (4.6%) | 27 (8.3%) | 19 (7.0%) | 18 (6.7%) |  |
| ISUP Grade at Prostatectomy |  |  |  |  |  |
| 1 | 118 (16%) | 16 (4.9%) | 13 (4.8%) | 15 (5.6%) |  |
| 2 | 335 (47%) | 127 (39%) | 128 (47%) | 110 (41%) |  |
| 3 | 178 (25%) | 103 (32%) | 75 (28%) | 80 (30%) |  |
| 4 | 46 (6.4%) | 52 (16%) | 25 (9.3%) | 44 (16%) |  |
| 5 | 41 (5.7%) | 29 (8.9%) | 29 (11%) | 21 (7.8%) |  |
| ISUP Concorance between SBx+TBx and prostatectomy |  |  |  |  |  |
| Concordant | 429 (60%) | 171 (52%) | 158 (59%) | 142 (53%) |  |
| Upstaged | 182 (25%) | 102 (31%) | 82 (30%) | 84 (31%) |  |
| Downgraded | 107 (15%) | 54 (17%) | 30 (11%) | 44 (16%) |  |
| ISUP Concorance between TBx and prostatectomy |  |  |  |  |  |
| Concordant | 371 (52%) | 156 (48%) | 148 (55%) | 130 (48%) |  |
| Upstaged | 277 (39%) | 130 (40%) | 99 (37%) | 105 (39%) |  |
| Downgraded | 70 (9.7%) | 41 (12%) | 23 (8.5%) | 35 (13%) |  |
| EAU risk group at SBx+TBx, n (%) |  |  |  |  |  |
| Low-risk | 156 (22%) | 33 (10%) | 36 (13%) | 30 (11%) |  |
| Intermediate risk | 409 (57%) | 202 (62%) | 159 (59%) | 171 (63%) |  |
| High-risk | 153 (21%) | 92 (28%) | 75 (28%) | 69 (26%) |  |
| EAU risk group at TBx, n (%): |  |  |  |  |  |
| Low-risk | 212 (30%) | 53 (16%) | 49 (18%) | 47 (17%) |  |
| Intermediate risk | 375 (52%) | 195 (60%) | 151 (56%) | 163 (60%) |  |
| High-risk | 131 (18%) | 79 (24%) | 70 (26%) | 60 (22%) |  |
| EAU risk group at prostatectomy, n (%): |  |  |  |  |  |
| Low-risk | 105 (15%) | 16 (4.9%) | 11 (4.1%) | 15 (5.6%) |  |
| Intermediate risk | 487 (68%) | 213 (65%) | 188 (70%) | 177 (66%) |  |
| High-risk | 126 (18%) | 98 (30%) | 71 (26%) | 78 (29%) |  |
| *SMD displayed only for variables included in the propensity-score match  TBx: Targeted Biopsy PSA: Prostate-Specific Antigen MRI: Magnetic-Resonance Imaging DRE: Digito-Rectal Examination  PIRADS: Prostate Imaging–Reporting and Data System ISUP: International Society of Urological Pathology SBx: Systematic Biopsy EAU: European Association of Urology | | | | | |

**Supplementary Table 2.** Cross-tabulation of highest International Society of Urological Pathology (ISUP) grade group found at Targeted Biopsy (TBx) and final grade at prostatectomy, stratified for TBx technique.

| ISUP Grade Group found at TBx: |  | ISUP Grade Group found at Prostatectomy: | | | | | |
| --- | --- | --- | --- | --- | --- | --- | --- |
| **Software-guided** |  | 1 | 2 | 3 | 4 | 5 | Total |
| No Cancer | n (%) | 41 (55%) | 14 (19%) | 11 (15%) | 5 (6%) | 3 (4%) | 74 |
| ISUP 1 | n (%) | 61 (36%) | 91 (54%) | 13 (8%) | 1  (1%) | 1  (1%) | 167 |
| ISUP 2 | n (%) | 12 (4%) | 199 (70%) | 69 (24%) | 4  (1%) | 1  (0.35%) | 285 |
| ISUP 3 | n (%) | 2 (2%) | 24 (23%) | 66 (63%) | 9  (8%) | 4  (4%) | 105 |
| ISUP 4 | n (%) | 1 (2%) | 7 (12%) | 18 (30%) | 23 (39%) | 10  (17%) | 59 |
| ISUP 5 | n (%) | 1 (4%) | 0 | 1  (4%) | 4 (14%) | 22  (79%) | 28 |
| Total | n (%) | 118 (16%) | 335 (47%) | 178 (25%) | 46 (6%) | 41  (6%) | 718 |
| **Cognitive** |  |  |  |  |  | | |
| 0 | Count | 4 (15%) | 10 (37%) | 8 (29%) | 4 (15%) | 1  (4%) | 27 |
| 1 | Count | 7 (18%) | 20 (51%) | 10 (26%) | 2  (5%) | 0 | 39 |
| 2 | Count | 2 (2%) | 79 (64%) | 34 (27%) | 8  (6%) | 1  (1%) | 124 |
| 3 | Count | 3 (4%) | 17 (22%) | 36 (47%) | 16 (21%) | 5  (6%) | 77 |
| 4 | Count | 0 | 1  (3%) | 9 (25%) | 19 (53%) | 7  (19%) | 36 |
| 5 | Count | 0 | 0 | 6  (25%) | 3 (13%) | 15  (63%) | 24 |
| Total | Count | 16 (5%) | 127 (39%) | 103 (31%) | 52 (16%) | 29  (9%) | 327 |

**Supplementary Table 3.** Multivariable logistic regression on International society of Urological Pathology concordance at radical prostatectomy in the matched cohort, compared to ISUP grade at targeted biopsy (TBx) and Systematic + Targeted Biopsy (SBx + TBx).

| **ISUP Grade Concordance at Prostatectomy, compared to ISUP at:** | **TBx** | | **SBx + TBx** | |
| --- | --- | --- | --- | --- |
|  | **HR (IC 95%)** | **P value** | **HR (IC 95%)** | **P value** |
| **Age**  **Previous Negative Biopsy**  (Biopsy naïve ref.)  **Suspicious/Positive DRE**  (Negative DRE ref.)  **PSA (ng/ml)**  **Prostate Volume**  **PIRADS 4**  **PIRADS 5**  (PIRADS 3 ref.)  **Transrectal Approach**  (Transperineal ref.)  **Cognitive TBx**  (Software-guided TBx ref.) | 0.99 (0.97-1.02)  0.91 (0.48-1.71)  0.89 (0.61-1.29)  1.01 (0.99-1.04)  1.00 (0.99-1.01)  1.26 (0.71-2.25)  1.13 (0.63-2.05)  0.97 (0.68-1.37)  0.76 (0.54-1.07) | 0.9  0.8  0.5  0.4  0.9  0.4  0.7  0.8  0.1 | 1.01 (0.98-1.03)  1.29 (0.68-2.50)   - 1. (0.69-1.48)   1.01 (0.99-1.03)  1.00 (0.99-1.01)  1.25 (0.70-2.24)  0.97 (0.53-1-76)  0.69 (0.48-0.98)  0.77 (0.55-1.09) | 0.6  0.4  0.9  0.4  0.7  0.4  0.9  **0.04**  0.1 |
| **DRE:** Digito-rectal examination **PSA:** Prostate-Specific Antigen **PIRADS:** Prostate Imaging Reporting And Data System | | | | |

**Supplementary Table 4.** Multivariable logistic regression on International society of Urological Pathology upgrade at radical prostatectomy in the matched cohort, compared to ISUP grade at targeted biopsy (TBx) and Systematic + Targeted Biopsy (SBx + TBx).

| **ISUP Grade Upstaging at Prostatectomy, compared to ISUP at:** | **TBx** | | **SBx + TBx** | |
| --- | --- | --- | --- | --- |
|  | **HR (IC 95%)** | **P value** | **HR (IC 95%)** | **P value** |
| **Age**  **Previous Negative Biopsy**  (Biopsy naïve ref.)  **Suspicious/Positive DRE**  (Negative DRE ref.)  **PSA (ng/ml)**  **Prostate Volume**  **PIRADS 4**  **PIRADS 5**  (PIRADS 3 ref.)  **Transrectal Approach**  (Transperineal ref.)  **Cognitive TBx**  (Software-guided TBx ref.) | 1.00 (0.98-1.03)  1.06 (0.53-2.05)  0.74 (0.50-1.12)  0.98 (0.96-1.01)  1.00 (0.99-1.01)  0.77 (0.43-1.43)  0.85 (0.46-1.59)  1.68 (1.15-2.46)  1.05 (0.73-1.53) | 0.9  0.3  0.6  0.4  0.5  0.4  0.6  0.7  0.5 | 1.00 (0.98-1.03)  1.06 (0.53-2.05)  0.74 (0.50-1.12)  0.98 (0.96-1.01)  1.00 (0.99-1.01)  0.77 (0.43-1.43)  0.85 (0.46-1.59)  1.68 (1.15-2.46)  1.05 (0.73-1.53) | 0.7  0.9  0.2  0.4  0.9  0.4  0.6  **0.01**  0.8 |
| **DRE:** Digito-rectal examination **PSA:** Prostate-Specific Antigen **PIRADS:** Prostate Imaging Reporting And Data System. | | | | |

**Supplementary Table 5.** Cross-tabulation of European Association of Urology (EAU) prostate cancer risk group at Targeted Biopsy (TBx) and final risk group at prostatectomy, stratified for TBx technique.

|  | | | | | | |
| --- | --- | --- | --- | --- | --- | --- |
| EAU Risk Group found at TBx: |  | EAU Risk Group found at Prostatectomy: | | | | |
|  |  |  | Low | Intermediate | High | Total |
| **Software-guided** | Low | n (%) | 92 (43%) | 114 (54%) | 6 (3%) | 212 |
|  | Intermediate | Count | 12 (3%) | 347 (93%) | 16 (4%) | 375 |
|  | High | Count | 1 (0.76%) | 26 (20%) | 104 (79%) | 131 |
|  | Total | Count | 105 (15%) | 487 (68%) | 126 (17%) | 718 |
| **Cognitive** | Low | Count | 11 (21%) | 36 (68%) | 6 (11%) | 53 |
|  | Intermediate | Count | 5 (3%) | 165 (85%) | 25 (13%) | 195 |
|  | High | Count | 0 | 12 (15%) | 67 (85%) | 79 |
|  | Total | Count | 16 (5%) | 213 (65%) | 98 (30%) | 327 |
